# Investigating thyroid function and iodine status in adolescents with and without paediatric major depressive disorder

**DOI:** 10.1017/S0007114524001910

**Published:** 2024-09-28

**Authors:** Ester Osuna, Jeannine Baumgartner, Andreas Walther, Sophie Emery, Mona Albermann, Noemi Baumgartner, Klaus Schmeck, Susanne Walitza, Michael Strumberger, Martin Hersberger, Michael B. Zimmermann, Isabelle Häberling, Gregor Berger, Isabelle Herter-Aeberli

**Affiliations:** 1 Laboratory of Human Nutrition, Institute of Food, Nutrition and Health, ETH Zürich, Zürich, Switzerland; 2 Department of Nutritional Sciences, King’s College London, London, UK; 3 Department of Clinical Psychology and Psychotherapy, University of Zurich, Zurich, Switzerland; 4 Department of Child and Adolescent Psychiatry and Psychotherapy, Psychiatric Hospital, University of Zurich, Zurich, Switzerland; 5 Department of Clinical Research, Medical Faculty, University of Basel, Basel, Switzerland; 6 Research Department of Child and Adolescent Psychiatry, Psychiatric University Hospitals Basel, University of Basel, Basel, Switzerland; 7 Division of Clinical Chemistry and Biochemistry, University Children’s Hospital Zurich, University of Zurich, Zurich, Switzerland; 8 Laboratory of Nutrition and Metabolic Epigenetics, Institute of Food, Nutrition and Health, ETH Zürich, Zürich 8092, Switzerland

**Keywords:** Adolescents, Hypothyroxinaemia, Iodine, Paediatric Major Depressive Disorder

## Abstract

Depression has been associated with subclinical hypothyroidism and altered hypothalamic-pituitary-thyroid axis functioning. Adequate iodine nutrition is essential for healthy thyroid functioning. We therefore determined associations of iodine and thyroid status with paediatric major depressive disorder (pMDD) among Swiss adolescents and explored whether associations are sex-specific and mediated by stress. We conducted a matched case–control study in 95 adolescents with diagnosed pMDD and 95 healthy controls. We assessed depression severity using the Children’s Depression Rating Scale-Revised and stress using the perceived stress scale (PSS) and measuring hair cortisol levels. We determined iodine status by measuring urinary iodine concentrations (UIC) and thyroid status by thyroid-stimulating hormone (TSH) and free thyroxine (FT4) in serum. Median (IQR) UIC did not differ between cases (121 (87, 174) µg/l) and controls (114 (66, 183) μg/l, *P* = 0·3). Median TSH and FT4 were lower in cases than controls (TSH: 1·36 (0·91, 2·00) mlU/l *v.* 1·50 (1·18, 2·06) mlU/l, *P* = 0·039; FT4: 14·7 (12·9, 16·9) pmol/l *v.* 15·7 (14·3, 17·2) pmol/l, *P* = 0·004). The prevalence of hypothyroxinaemia (normal TSH; low FT4) was higher among female cases than controls (21 % *v.* 4%, *P* = 0·006). PSS scores were higher while hair cortisol was lower in cases than controls (PSS: 25 (20, 28) *v.* 11 (7, 15), *P* < 0·001; cortisol: 2·50 (1·34, 3·57) pg/mg *v.* 3·23 (1·79, 4·43) pg/mg, *P* = 0·044). After adjusting for confounders, the associations of TSH and hair cortisol with pMDD were no longer significant. Furthermore, TSH and FT4 were not associated with PSS scores and hair cortisol levels. Summarising, iodine nutrition was adequate for adolescents with and without pMDD. However, FT4 concentrations were lower in those with pMDD, and 1 in 5 female adolescents with pMDD were hypothyroxinaemic.

Over 300 million people worldwide are affected by depression^(1)^, which makes depression a leading cause of disability worldwide^(2)^. Depression during adolescence is associated with poor educational, work and social functioning as well as an increased rate of smoking, substance abuse, eating disorders and obesity^(3)^. Also, early onset of depression is a risk factor for chronic and recurrent forms of depression in adulthood^(4)^. Nevertheless, paediatric major depressive disorder (pMDD) often remains undiagnosed and therefore untreated^(5)^. Experts estimate that 11 % of children and adolescents have an episode of pMDD before reaching adulthood^(6)^, making it one of the most common psychiatric disorders during childhood and adolescence^(7)^. It is important to better understand its aetiology to develop effective strategies to prevent pMDD or delay its progression.

Iodine deficiency results in inadequate production of thyroid hormones since iodine is an essential component of the thyroid hormones thyroxine and triiodothyronine. In the foetus, inadequate thyroid hormones as a result of inadequate maternal iodine intake can impair myelination, cell migration, differentiation and maturation in the brain^(8,9)^. The aetiology of depression remains unclear, but it is most likely multifactorial. For instance, a large body of evidence indicates that the hypothalamic-pituitary-thyroid (HPT) axis is altered in various psychiatric disorders such as bipolar disorder, schizophrenia, anxiety disorders and depression^(10–12)^. Potential mechanisms of the HPT axis being involved in the aetiology of depression include reduced availability of thyroid hormones implicating decreased myelination and synaptic plasticity within the brain^(13)^. Both decreased myelination and synaptic plasticity have been associated with depression before^(14,15)^. Furthermore, there is evidence that factors such as age and sex might play a role in the link between thyroid dysfunction and depression^(16,17)^. Nevertheless, these previous studies and meta-analyses fail to consistently establish a link between pathological thyroid function and depressive disorders^(16,17)^. To date, most of the studies linking pathological thyroid function, such as subclinical hypothyroidism, with depressive disorders have been conducted in adults. However, findings from studies in adults cannot be generalised to adolescents due to differences in the developmental stage and nutrient requirements, as well as poorer diet quality in this age group^(18,19)^. Therefore, it seems important to investigate the link between iodine nutrition, the HPT axis and depression among adolescents to gain further insights into the aetiology of this disorder.

There is evidence from animal models and human studies that an altered hypothalamic-pituitary adrenal (HPA) axis might affect the production of thyrotropin-releasing hormone^(20,21)^. Environmental stress is known to increase the sensitivity of the HPA axis and enhances response to subsequent stressors^(22,23)^. Furthermore, there is robust evidence for the HPA axis being implicated in the pathophysiology of MDD and other stress-related diseases by alterations of the endocrine system in adults^(22,24)^. Nevertheless, there is still a lack of evidence for a relationship between the HPA and HPT axis in depressed adolescents^(25)^.

Around 10 % of adolescents in Switzerland experience periodic depressive symptoms^(26)^. Moreover, even though Switzerland has a model salt iodisation programme, and school-aged children were shown to have adequate iodine intakes^(27,28)^, national studies report poor iodine nutrition in adults^(29)^ and pregnant women^(28)^. Thus, the aim of this study was to determine associations of iodine status and thyroid function with pMDD in Swiss adolescents. We further explored whether these associations may be mediated by stress (measured as perceived stress and cortisol levels). Finally, we investigated whether these associations are sex-specific and related to antidepressant use. We hypothesised that adolescents with pMDD have a higher prevalence of hypothyroidism or subclinical hypothyroidism compared to healthy adolescents without pMDD and that these differences are related to differences in iodine nutrition. Further, we hypothesised aberrant thyroid hormone concentrations to be associated with stress, which in turn, were hypothesised to be associated with pMDD.

## Participants and methods

### Study design

This study is an observational case–control study in adolescents with diagnosed pMDD and healthy controls aged 13–17 years. The cases and controls were matched according to sex, age group (13 to < 16 and 16 to < 18 years) and educational level in a 1:1 ratio. To calculate the sample size, G * Power V3.1.9.2 was used. A power calculation was applied to a logistic model where the Children’s Depression Rating Scale-Revised (CDRS-R, described below) score was coded as a dichotomous variable in a model with 10 covariates (with a residual (R^2^ = 0·2). In this model, one sd increase of the continuous predictor generated an OR of 1·5 and 2. According to these power calculations, a sample size of 200 individuals with a 1:1 matching ratio between cases and controls was sufficient (power > 80 %, beta ≤ 20 %) to detect medium to large effect sizes for a type-I error of 5 % (*α* = 0·05). Up to a 10 % dropout rate, these results seemed robust. To have a balanced study population, we aimed to include 102 cases and 102 controls, with equal representation of sex, age groups and educational level in cases and controls. In the first age group of 13 to < 16 years of age, the aim was to recruit 50 female and male participants (25 each) in lower secondary school. In the second age group of 16 to < 18 years, the aim was to recruit 52 female and male participants: 13 female and male participants each for (1) vocational education and (2) baccalaureat/vocational baccalaureat at higher secondary school. This recruitment strategy was applied to recruit the controls of this case–control study. Afterwards, the cases were randomly selected to match the controls according to sex, age group and educational level.

The ethics committee of the Canton of Zurich approved this study (BASEC-Nr. 2019-00717), and it was registered at www.ClinicalTrials.gov (NCT04158869). This study is an add-on study to the investigator-initiated clinical trial (SNSF 33IC30_166826, BASEC-Nr. 2016-02116). Caregivers and adolescents ≥ 14 years of age consented to this study in written form, and adolescents < 14 years of age consented orally before any research-related assessments were conducted.

This manuscript is one of a series of papers investigating potential nutritional factors involved in the aetiology of pMDD^(30,31)^.

### Participants and procedures

#### Control group

The control group was recruited and assessed by a study team of the Laboratory of Human Nutrition at ETH Zurich, Switzerland. From September 2019 until December 2020, healthy female and male controls were recruited from the canton of Zurich and surrounding German-speaking cantons of Switzerland. Recruiting of controls took place in schools, leisure time clubs and via social media. Inclusion criteria for controls were age of 13 to < 18 years; no present nor past primary diagnosed psychiatric disorder according to the Mini-International Neuropsychiatric Interview for Children and Adolescents^(32)^; and no use of chronic medication. Controls were not eligible if they were unable to follow the study procedures, for example due to language barriers; if they took *n*-3 PUFA supplements (providing > 600 mg combined EPA/DHA) for more than four weeks within the last six months; and if they reported pre-existing neurological or medical conditions likely to result in the development of depressive symptoms. After consenting and enrolling into the study, participants electronically completed questionnaires on REDCap® (Research Electronic Data Capture) within two weeks prior to the physical data assessment at ETH Zurich.

#### Paediatric major depressive disorder group

The cases in this study were participants of the *Omega*-*3 Fatty Acids as treatment for Paediatric Major Depressive Disorder Trial (Omega*-*3 pMDD)* under the lead of the Department of Child and Adolescent Psychiatry and Psychotherapy of the Psychiatric Hospital, University of Zurich, which were randomly selected to match the controls. The protocol of the Omega-3 pMDD study has been published previously^(33)^. The recruitment of participants took place in seven different i*n*- and outpatient services in five German-speaking cantons of Switzerland from May 2017 until June 2021. The adolescents were either informed about the study by their clinicians in one of the participating centres or contacted the study team on their own initiative after seeing posters or flyers. The inclusion criteria for the cases were for teenagers aged between 13 and < 18 years and a main diagnosis of MDD according to DSM-IV criteria^(34)^ of at least moderate severity defined by a CDRS-R^(35)^ total score of ≥ 40. Cases were not eligible if they fulfilled diagnostic criteria for an eating disorder within the last 6 months or a lifetime diagnosis of schizophrenia, bipolar affective disorder, substance use dependency, mental retardation or pervasive development disorder. Further, cases were not eligible if they had pre-existing neurological or medical conditions likely causing their depressive symptoms; if they were taking *n*-3 PUFA supplementation (> 600 mg combined EPA/DHA) within the last 6 months; or if their families were unable to follow the study procedures, for example, due to language barriers. After consenting to the study, the screening interview was conducted with the adolescent and a parent separately. The inclusion and exclusion criteria were assessed with the Kiddie-Schedule for Affective Disorders and Schizophrenia^(36)^ for assessing the presence of MDD and the CDRS-R for assessing the severity of the depression. For this case–control study, only data (biological samples and CDRS-R scores) from the baseline interview before randomisation were used.

### Data collection

This study tried to align the procedures between cases and controls as much as possible.

#### Assessment of anthropometry and socio-demographic information

For the cases and the controls, weight (to the nearest 0·1 kg) and height (to the nearest 0·5 cm) were measured at the end of the physical data assessment. The BMI was calculated using the individual’s weight in kilograms (kg) divided by the individual’s height in metres (m) squared (kg/m^2^). BMI-for-age z-scores were calculated using the R package ‘anthroplus’ provided by the WHO. Further, the data on BMI were then categorised into four BMI categories of underweight, normal weight, overweight and obese according to the WHO’s age-dependent cut-offs^(37)^. In adolescents, a z-score < –1 sd coincides with adult underweight (BMI < 18), a z-score > +1 sd coincides with adult overweight (BMI ≥ 25) and a z-score > +2 with adult obesity (BMI ≥ 30). Socio-demographic and socio-economic data were assessed using self-reported questionnaires which the participants were asked to fill out together with one parent.

#### Assessment of depression severity and perceived stress

The CDRS-R was used to assess depression severity. The CDRS-R is a semi-structured clinical interview which takes 15–20 min to administer. It is one of the most frequently used rating scales for measuring the severity of depression and change in depressive symptoms in children and adolescents^(38)^. The validity of the scale has been shown for adolescents^(38)^ and children^(39)^. By providing the possibility to conduct the interview with the child, the parents and/or teacher, the interview allows a comprehensive assessment of the severity of the child’s or adolescent’s depression. The interview covers 17 depressive symptoms rated on a 5- to 7-point Likert scale. The domains of depressive symptoms are aligned with the DSM-IV criteria for childhood depression and cover suicidal ideation, social withdrawal, sleep disturbance, excessive fatigue, etc. Individuals are asked about information on 14 items, and three non-verbal symptoms are assessed only by the interviewer (depressed facial affect, hypoactivity and speech velocity). The interviewers were trained to conduct the interview. The individual ratings were summed up to a total score ranging from 17 to 113 with a score of ≥ 40 being used as a cut-off for pMDD^(40)^.

Data on perceived stress were assessed with a self-reporting Perceived Stress Scale (PSS)^(41)^. This questionnaire is structured into 10 items which are rated on a 0- to 4-point Likert scale with total scores ranging from 0 to 40. Higher scores indicate that an individual perceives their current situation as more stressful. The validity of the scale’s German version has been shown in a German female and male population in the age range from 14 to 90 years^(42)^.

#### Biochemical analysis

Participants were asked to collect a spot urine sample (Urin-Monovette®, Sarstedt) voluntarily to assess the urinary iodine concentration (UIC) as a marker for iodine status. No standardised collection timepoint was used. The UIC was measured using the Pino modification of the Sandell-Kolthoff reaction^(43)^. UIC in spot urine samples was measured by one single person at the Laboratory for Human Nutrition, which successfully participates in the EQUIP network (Ensuring the Quality of Urinary Iodine Procedures, USA Centers for Disease Control and Prevention, Atlanta, GA). All urine samples were measured in duplicate and re-analysed when the difference in absorbance was > 5 % for UIC > 150 µg/l, > 10 % for UIC 50–150 µg/l and > 15 % for UIC < 50 µg/l. Control urine samples were added to every plate for external quality control. The reference range for adequate iodine nutrition was set according to WHO reference values for UIC between 100–199 μg/l^(44)^.

Three hair strands with a minimum of 20 mg were cut as close as possible to the scalp from the posterior vertex. For controls, hair strands were taken on the assessment day. For the cases, hair samples were taken 6 weeks into the intervention. Samples were processed and analysed at the Dresden LabService GmbH (Tatzberg, 47, 01307, Dresden, Germany). The procedure of glucocorticoid analysis has been published before^(45)^. In brief, the samples were cut into 3 cm segments obtained from the scalp-near site, which represent the integrated hormone concentration over the last 3 months. This is due to the fact that on average hair growth rate is 1 cm per month^(46)^. To provide an estimate of cumulative hormone concentration for two six-week periods, the 3 cm segments were cut into two 1·5 cm segments^(47,48)^. For controls, the 1·5 cm closest to the scalp was analysed to compare to the blood samples collected on the same day. In cases, the segment 1·5 cm away from the scalp was used to account for the 6 weeks of time difference between blood and hair collection. Biochemical analysis was conducted using liquid chromatography coupled with tandem MS^(49)^.

Venous blood was drawn into serum tubes (BD Vacutainer) and was let stand for min. 60 min to allow clotting. Afterwards, the serum tubes were centrifuged, and the serum was then stored at −80°C until further analysis. The thyroid parameters FT4 and thyroid-stimulating hormone (TSH) were measured in serum by electrochemiluminescence (Cobas e411, Roche Elecsys) at the University Children’s Hospital Zurich, Switzerland. The reference range for peripheral FT4 in the age group of > 11 to < 20 years of age was defined as FT4 values between 12·6 pmol/l to 21 pmol/l^(50)^. The reference range for peripheral TSH values in the age group of > 11 to < 20 years of age was defined as TSH values between 0·51 mlU/l to 4·3 mlU/l^(51)^. Values outside the reference range were classified accordingly. Clinical or overt hypothyroidism was characterised as FT4 < 12·6 pmol/l and TSH > 4·3 mlU/l. Subclinical hypothyroidism was characterised as normal FT4 and TSH > 4·3 mlU/l. Clinical or overt hyperthyroidism was defined by FT4 > 21 pmol/l and TSH < 0·51 mlU/l. Subclinical hyperthyroidism was characterised by normal FT4 and TSH < 0·51 mlU/l. Hypothyroxinaemia was characterised by FT4 < 12·6 pmol/l and normal TSH values.

### Data management and statistical methods

Data capture for the controls was done either electronically on REDCap® or on paper and retroactively entered into the REDCap® system. When captured on paper, the person assessing the data entered it into the system and later the data was checked for entry errors by a second member of the study team. REDCap® is a secure, web-based software platform and electronic data capture tool designed for research studies which is hosted at ETH Zurich^(52,53)^. For the cases, data were assessed on paper and entered into the electronical data capture tool secuTRIAL by two individual persons. Data entry was checked by a third person for entry errors. After completing the matching between the cases and the controls, all data were combined and managed using REDCap®.

Data processing and statistical analysis of data were performed using R Version 3.6.0. By means of Q-Q plots, histograms and Shapiro-Wilk test, data were tested for outliers and normality. Normally distributed data and non-normally distributed data were expressed as mean (sd) and as medians (interquartile range), respectively. Wilcoxon rank sum test was applied to test not normally distributed continuous data and *t* test for normally distributed data. Chi-square tests were applied to assess significant differences when the expected cell count was ≥ 5 and Fisher’s exact test when the expected cell count was < 5. For producing tables and calculating these differences, the R package ‘gtsummary’ was used^(54)^. Further, to assess associations of different thyroid function indicators and indicators of stress with depression (CDRS-R score ≥ 40), unconditional multiple logistic regression analysis was performed using two different statistical models. In model 1, the covariates of the matching criteria sex, age, educational level and BMI-for-age z-scores were included. In model 2, the use of antidepressants was additionally included as a covariate since there is data indicating a possible association between antidepressant use and thyroid function^(55)^. To assess associations of the different thyroid function indicators with PSS scores and hair cortisol levels, unconditional multiple linear regression analysis was performed, using the same models.

## Results

**Fig. 1.**
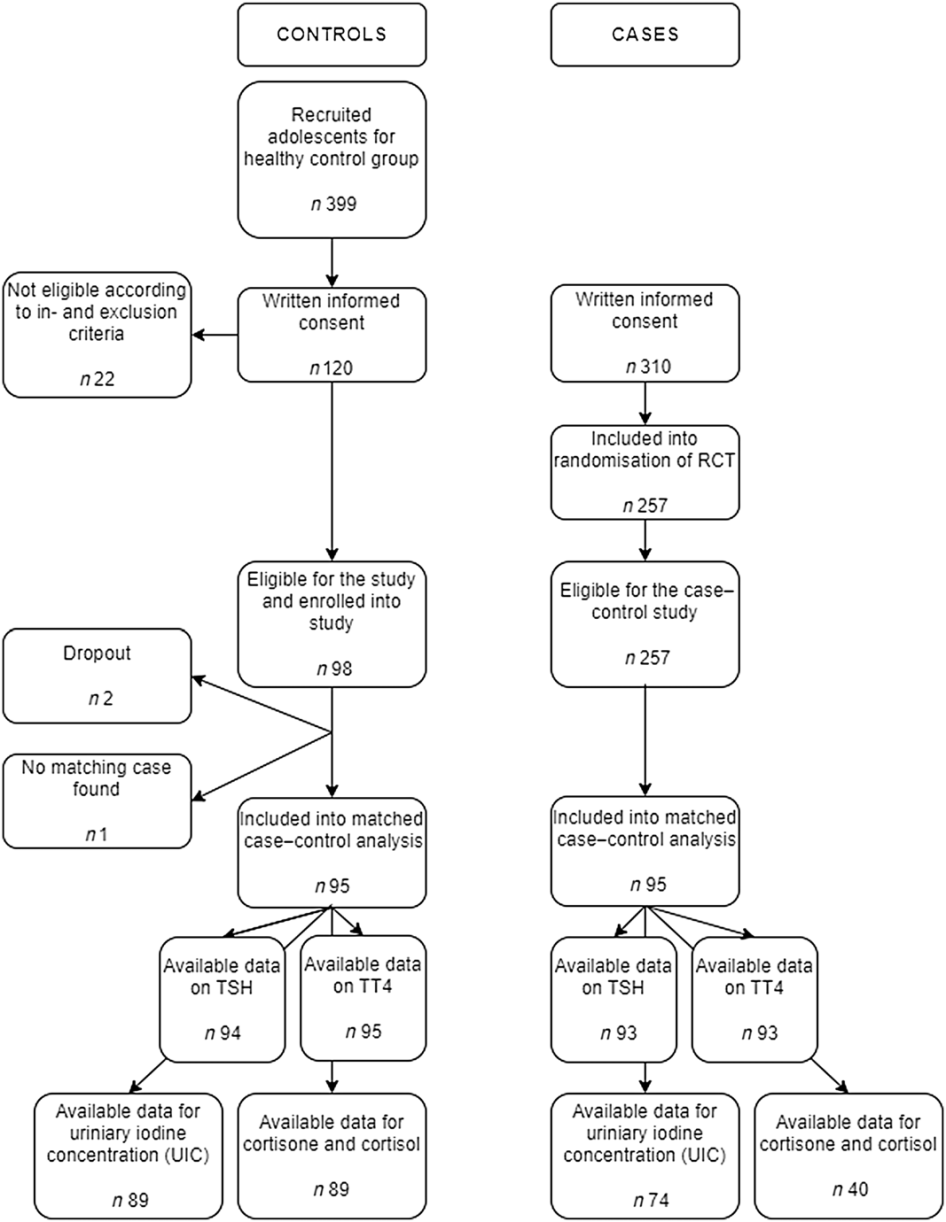
Flow chart of this case–control study’s included adolescents with and without diagnosed paediatric major disorder (pMDD). RCT: Randomised Control Trial; TSH: thyroid-stimulating hormone; FT4: thyroxine.

After the recruitment process, 98 controls were enrolled into this case–control study. Thereof, two individuals dropped out: one voluntarily after the screening interview and one by not providing a blood sample. For the selection of the cases from the Omega-3 pMDD study, a total of 257 participants were randomised to one of the two treatment arms and were therefore eligible as cases for the case–control study. One control could not be matched to a case according to the matching criteria. Therefore, a total of 95 controls were matched to 95 cases according to sex, age group (13 to < 16 and 16 to < 18) and educational level. The final sample size for this case–control analysis was *n* 190. A detailed flow chart of the study inclusion can be seen in Fig. 1. For four adolescent pairs in the age group 16 to < 18 years of age, the matching was done only according to sex and age group since matching by educational level was not possible.

The detailed participant characteristics are presented in Table 1. The matching between cases and controls was successful, which was shown by the absence of significant differences in age, sex and educational level. Also, there were no significant differences between cases and controls with respect to BMI-for-age z-scores. On the other hand, significantly higher CDRS-R and PSS scores were observed among cases compared to controls (both *P* < 0·001). Among cases, 22 % of adolescents had a recurrent episode of depression and 38 % used antidepressant drugs at study inclusion. The use of antidepressants at study inclusion correlated with the recurrence of episodes (χ^2*s*^ = 15·6, *P* < 0·001).

**Table 1.** Characteristics of Swiss adolescents with (*n* 95) and without (*n* 95) paediatric major depressive disorder (pMDD) (Median values and interquartile ranges; mean values and sd; numbers and percentages)

	Overall		Cases		Controls		*P*-value
	*n*	%	*n*	%	*n*	%	
Age							
Median	16·1		16·1		16·0		0·8^‡^
IQR	14·9, 17·1		14·9, 17·2		14·9, 17·1		
Sex							> 0·9^§^
Female	110	58 %	55	58 %	55	58 %	
Male	80	42 %	40	42 %	40	42 %	
CDRS score							
Median	36		56		18		**< 0·001^‡^**
IQR	18, 56		50, 62		17, 20		
PSS score*							
Median	17		25		11		**< 0·001^‡^**
IQR	11, 25		20, 28		7, 15		
BMI-for-age z-score^†^							
Mean	0·20		0·25		0·16		0·4^‡^
sd	1·03		1·05		1·02		
BMI category^†^							0·5^||^
Underweight	7	4 %	2	2 %	5	5 %	
Normal weight	138	76 %	65	75 %	73	77 %	
Overweight	20	11 %	12	14 %	8	8 %	
Obese	17	9 %	8	9 %	9	10 %	
Ethnicity							0·051^||^
European	174	92 %	87	92 %	87	92 %	
East-Asian	5	3 %	5	5 %	0	0 %	
Indian-Asian	2	1 %	0	0 %	2	2 %	
Middle East	2	1 %	1	1 %	1	1 %	
Not declared	7	4 %	2	2 %	5	5 %	
Swiss educational levels							0·8^§^
Lower secondary school (Mandatory school)	104	55 %	54	57 %	50	53 %	
Upper secondary school							
Vocational education	28	15 %	14	15 %	14	15 %	
Baccalaureat/ vocational baccalaureat	58	31 %	27	28 %	31	33 %	
Participants using antidepressants at study inclusion	36	19 %	36	38 %	0	0 %	**< 0·001^§^**
Participants with recurrent episode at study inclusion	22	12 %	22	23 %	0	0 %	**< 0·001^§^**
Participants with recurrent episode AND using antidepressants	11	6 %	11	12 %	0	0 %	**< 0·001^§^**

CDRS: Children’s depression rating scale; PSS: perceived stress scale; IQR: interquartile range

Median (IQR); Mean (sd), *n* (%).

*P*-values in bold were statistically significant.

* Data on PSS score were not available for all participants (*n*_cases_ = 93; *n*_controls_ = 95).

† Data on BMI were not available for all participants (*n*_cases_ = 87; *n*_controls_ = 95), BMI-for-age z-scores and BMI categories were defined according to WHO reference data (Adolescents with a z-score at < –1 sd coincide with adult underweight (BMI < 18), adolescents with a z-score > +1 sd coincide with adult overweight (BMI = 25) and adolescents with a z-score > +2 with adult obesity (BMI = 30)).

‡ Wilcoxon rank sum test;

§ Pearson’s chi-squared test;

|| Fisher’s exact test.

A description of iodine and thyroid status parameters and glucocorticoids is displayed in Table 2. Urine samples were not available for 27 participants and hair samples for 61 participants, since the collection was not compulsory. Sufficient blood samples for TSH and FT4 analysis were not available for three of the study participants. The median (interquartile range) UIC for the entire study population was 119 (75, 178) μg/l, with no significant difference between groups (*P* = 0·3). However, TSH and FT4 values were significantly lower among cases compared to controls (TSH: 1·36 (0·91, 2·00) mlU/l *v.* 1·50 (1·18, 2·06) mlU/l, respectively, *P* = 0·039; and FT4: 14·7(12·9, 16·9) pmol/l *v.* 15·7 (14·3, 17·2) pmol/l, respectively, *P* = 0·004). There was a significantly higher prevalence of thyroid dysfunction among cases compared to controls of 17 % *v.* 7% (*P* = 0·039). The main type of thyroid dysfunction was attributed to a higher prevalence of hypothyroxinaemia among cases compared to controls (*P* = 0·012). Cases had lower hair cortisol levels compared to controls (2·50 (1·34, 3·57) *v.* 3·23 (1·79, 4·43), *P* = 0·044). In Table 3, iodine and thyroid status parameters are displayed by sex. Significantly lower FT4 values were observed among female cases compared to female controls (14·4 (12·6, 15·3) pmol/l *v.* 15·1 (14·2, 16·6) pmol/l, respectively, *P* = 0·004), but no difference was observed among males. Also, a significantly higher prevalence of thyroid dysfunction was observed among female cases compared to female controls with 21% *v.* 4%, respectively (*P* = 0·006), which was not observed among males. Finally, a trend for lower cortisol levels among female cases compared to female controls could be observed (2·62 (1·61, 3·49) pg/mg *v.* 3·69 (1·67, 4·96) pg/mg, respectively, *P* = 0·051).

**Table 2.** Summary of iodine and thyroid status indicators and hair glucocorticoids in a sample of adolescents with and without diagnosed paediatric major depressive disorder (pMDD) (Median values and interquartile ranges; numbers and percentages)

	Overall		*n*_cases_	Cases		*n*_controls_	Controls		*P*-value
	Median	IQR		Median	IQR		Median	IQR	
UIC (µg/l)	119	75, 178	74	121	87, 174	89	114	66, 183	0·3^||^
TSH (mIU/l)	1·45	1·02, 2·01	93	1·36	0·91, 2·00	94	1·50	1·18, 2·06	**0·039^||^**
T4 (pmol/l)	15·1	13·8, 17·0	93	14·7	12·9, 16·9	95	15·7	14·3, 17·2	**0·004^||^**
	*n*	%		*n*	%		*n*	%	
Thyroid dysfunction*	23	12 %	92	16	17 %	94	7	7 %	0·039^**^
Types of thyroid dysfunction									
Subclinical hyperthyroidism^†^	2	1 %		2	2 %		0	0 %	
Hypothyroxinaemia^‡^	18	10 %		14	15 %		4	4 %	
Hyperthyroxinaemia^§^	3	2 %		0	0 %		3	3 %	
	Median	IQR		Median	IQR		Median	IQR	
Hair cortisol (pg/mg)	3·10	1·72, 4·30	40	2·50	1·34, 3·57	89	3·23	1·79, 4·43	**0·044^||^**
Hair cortisone (pg/mg)	6·07	3·87, 9·44	40	7·00	3·49, 11·12	89	5·63	3·98, 8·28	0·2^||^

UIC: urinary iodine concentration; TSH: thyroid-stimulating hormone; T4: thyroxine.

Median (Interquartile range (IQR)), *n* (%).

*P*-values in bold were statistically significant.

* Normal thyroid function in adolescents > 11 and < 20 years classified as TSH between 0·51–4·3 mIU/l and T4 between 12·6–21 pmol/l.

† Subclinical hyperthyroidism defined as normal T4 levels and TSH < 0·51 mlU/l.

‡ Hypothyroxinaemia defined as normal TSH levels and T4 < 12·6 pmol/l.

§ Hyperthyroxinaemia defined as normal TSH levels and T4 > 21 pmol/l.

|| Wilcoxon rank sum test;

** Pearson’s chi-squared test.

**Table 3.** Summary of iodine and thyroid status indicators and hair glucocorticoids by sex in a sample of adolescents with diagnosed paediatric major depressive disorder (pMDD) and without pMDD (Median values and interquartile ranges; numbers and percentages)

	Female							Male						
Characteristic	*n*_cases_	Cases		*n*_controls_	Controls		*P*-value	*n*_cases_	Cases		*n*_controls_	Controls		*P*-value
		Median	IQR		Median	IQR			Median	IQR		Median	IQR	
PSS score	55	25	22, 30	55	11	8, 15	**< 0·001^||^**	40	23·	16·5, 27	40	10·5	6, 13	**< 0·001^||^**
UIC (µg/l)	44	126	89, 174	52	107	51 183	0·2^||^	30	119	79, 170	37	128	75, 179	> 0·9^||^
TSH (mIU/l)	53	1·20	0·94, 1·90	55	1·42	1·02, 1·90	0·2^||^	40	1·45	0·87, 2·09	39	1·79	1·39, 2·20	0·1^||^
T4 (pmol/l)	52	14·4	12·6, 15·3	55	15·1	14·2, 16·6	**0·004^||^**	40	15·5	13·8, 17·6	40	16·3	14·9, 18·0	0·2^||^
		*n*	%		*n*	%			*n*	%		*n*	%	
Thyroid dysfunction*	52	11	21 %	55	2	4 %	**0·006^¶^**	40	5	12 %	39	5	13 %	> 0·9^¶^
Types of thyroid dysfunction														
Subclinical hyperthyroidism^†^		0	0 %		0	0 %			2	5 %		0	0 %	
Hypothyroxinaemia^‡^		11	21 %		2	4 %			3	8 %		2	5 %	
Hyperthyroxinaemia^§^		0	0 %		0	0 %			0	0 %		3	8 %	
		Median	IQR		Median	IQR			Median	IQR		Median	IQR	
Hair cortisol (pg/mg)	22	2·62	1·61, 3·49	53	3·69	1·67, 4·96	0·051^||^	18	2·49	1·12, 3·56	36	2·85	1·95, 4·18	0·4**
Hair cortisone (pg/mg)	22	6·43	2·96, 9·35	53	5·46	3·60, 7·73	0·8^||^	18	9·33	5·57, 12·6	36	5·96	4·52, 8·51	0·11**

UIC: urinary iodine concentration; TSH: thyroid-stimulating hormone; T4: thyroxine; PSS: perceived stress scale.

Median (interquartile range (IQR)), *n* (%).

*P*-values in bold were statistically significant.

* Normal thyroid function in adolescents > 11 and < 20 years classified as TSH between 0·51–4·3 mIU/l and T4 between 12·6–21 pmol/l.

† Subclinical hyperthyroidism defined as normal T4 levels and TSH < 0·51 mlU/l.

‡ Hypothyroxinaemia defined as normal TSH levels and T4 < 12·6 pmol/l.

§ Hyperthyroxinaemia defined as normal TSH levels and T4 > 21 pmol/l.

|| Wilcoxon rank sum test;

¶ Pearson’s chi-squared test;

** Wilcoxon rank sum exact test.

Table 4 shows iodine, thyroid status parameters and glucocorticoids of the cases by use of antidepressants. We observed a trend for higher UIC and lower TSH values among cases with antidepressant use (both *P* = 0·052).

**Table 4.** Summary of iodine and thyroid status indicators and hair glucocorticoids by antidepressant use in adolescents with diagnosed paediatric major depressive disorder (pMDD) (Median values and interquartile ranges; numbers and percentages)

	Cases					
	*n*	Use of antidepressants (*n* 36)		No use of antidepressants (*n* 59)		*P*-value
		Median	IQR	Median	IQR	
UIC (µg/l)	74	144	118, 180	110	77, 155	0·052^||^
TSH (mIU/l)	93	1·11	0·87, 1·71	1·39	1·13, 2·02	0·052^||^
T4 (pmol/l)	93	14·8	13·0, 17·0	14·6	13·0, 16·4	> 0·9^||^
		*n*	%	*n*	%	
Thyroid dysfunction*	92	6	18 %	10	17 %	> 0·9^¶^
Types of thyroid dysfunction						
Subclinical hyperthyroidism^†^		2	6 %	0	0 %	
Hypothyroxinaemia^‡^		4	12 %	10	17 %	
		Median	IQR	Median	IQR	
Hair cortisol (pg/mg)	40	3·15	1·86, 3·58	2·41	0·93, 3·36	0·4^||^
Hair cortisone (pg/mg)	40	7·24	5·73, 10·9	6·79	3·33, 12·0	0·6^||^

UIC: urinary iodine concentration; TSH: thyroid-stimulating hormone; T4: thyroxine.

Median (interquartile range (IQR)), *n* (%).

*P*-values in bold were statistically significant.

* Normal thyroid function in adolescents > 11 and < 20 years classified as TSH between 0·51–4·3 mIU/l and T4 between 12·6–21 pmol/l.

† Subclinical hyperthyroidism defined as normal T4 levels and TSH < 0·51 mlU/l.

‡ Hypothyroxinaemia defined as normal TSH levels and T4 < 12·6 pmol/l.

|| Wilcoxon rank sum test;

¶ Pearson’s chi-squared test.

A summary of conducted unconditional multiple logistic regression models assessing associations of iodine and thyroid status indicators as well as perceived stress and hair cortisol levels with pMDD is shown in Table 5. There was no significant association between UIC and pMDD. Higher TSH values were associated with lower odds for depression in the model controlling for sex, age, educational level and BMI-for-age z-scores (OR = 0·59 (0·36–0·93), *P* = 0·026), but this association was no longer significant when additionally adjusting for antidepressant use (OR = 0·75 (0·44–1·27), *P* = 0·3). Higher FT4 levels were associated with lower odds for depression (OR = 0·80 (0·67–0·94), *P* = 0·009) in both models. Higher PSS scores were also associated with higher odds for depression (OR = 1·38(1·26–1·55), *P* < 0·001) in both models. Higher hair cortisol levels were associated with lower odds for depression in model 1 (OR = 0·80 (0·65–0·96), *P* = 0·029), but this association was no longer significant when adjusting for antidepressant use (OR = 0·80 (0·61–0·98), *P* = 0·070). Table 6 shows a summary of unconditional multiple linear regression models assessing associations of iodine and thyroid status indicators with PSS scores and hair cortisol levels. No significant associations were found between iodine or thyroid status indicators and PSS scores. However, there was a trend for FT4 concentrations being negatively associated with PSS scores (beta = –0·49 (95 % CI: –1·00, 0·03), *P* = 0·065) when not adjusting for antidepressant use. Furthermore, there was a trend for PPS scores being negatively associated with hair cortisol (beta = –0·06 (95 % CI: –0·13, 0·00), *P* = 0·065).

**Table 5.** Unconditional multiple logistic regression models assessing associations of iodine and thyroid status indicators as well as perceived stress scale (PSS) scores and hair cortisol with paediatric major depressive disorder (pMDD) in Swiss adolescents (*n* 186)

	Model I*			Model II^†^		
	OR	lower 95 % OR–upper 95 % OR	*P*-value	OR	lower 95 % OR–upper 95 % OR	*P*-value
UIC (µg/l)	1·00	1·00–1·01	0·3	1·00	1·00–1·01	0·8
TSH (mIU/l)	0·59	0·36–0·93	**0·026**	0·75	0·44–1·27	0·3
FT4 (pmol/l)	0·81	0·70–0·92	**0·002**	0·80	0·67–0·94	**0·009**
PSS scores	1·42	1·30–1·59	**< 0·001**	1·38	1·26–1·55	**< 0·001**
Hair cortisol (pg/mg)	0·80	0·65–0·96	**0·029**	0·80	0·61–0·98	0·070

UIC: urinary iodine concentration; TSH: thyroid-stimulating hormone; T4: thyroxine; PSS: perceived stress scale.

*P*-values in bold were statistically significant.

The dependent variable was the diagnosis of depression (CDRS-R ≥ 40). The independent variables were iodine/thyroid status indicators, perceived stress and hair cortisol.

* Model I: controlled for sex, age, educational level and BMI-for-age z-scores.

† Model II: controlled for sex, age, educational level, BMI-for-age z-scores and antidepressant use.

**Table 6.** Unconditional multiple linear regression models assessing associations of iodine and thyroid status indicators with perceived stress scale (PSS) scores and hair cortisol (pg/mg) in Swiss adolescents (*n* 186) (95 % CI)

	Model I*						Model II^†^					
	R^2^	Adjusted R^2^	Beta	95 % CI	Standardised beta	*P*-value	R^2^	Adjusted R^2^	Beta	95 % CI	Standardised beta	*P*-value
PSS score												
UIC (µg/l)	0·094	0·058	0·01	–0·01, 0·03	0·06	0·5	0·250	0·214	0·00	–0·02, 0·02	–0·01	> 0·9
TSH (mIU/l)	0·090	0·057	–1·57	–3·4, 0·28	–0·13	0·1	0·230	0·198	–0·59	–2·3, 1·1	–0·05	0·5
FT4 (pmol/l)	0·095	0·063	–0·49	–1·0, 0·03	–0·14	0·065	0·236	0·205	–0·30	–0·79, 0·18	–0·09	0·2
Hair cortisol												
UIC (µg/l)	0·012	–0·045	0·00	–0·01, 0·00	–0·10	0·4	0·024	–0·043	0·00	–0·01, 0·00	–0·10	0·3
TSH (mIU/l)	0·025	–0·025	–0·54	–1·5, 0·40	–0·11	0·3	0·040	–0·018	–0·65	–1·6, 0·31	–0·13	0·2
FT4 (pmol/l)	0·018	–0·031	0·10	–0·17, 0·36	0·07	0·5	0·028	–0·030	0·09	–0·18, 0·35	0·06	0·5
PSS scores	0·041	–0·008	–0·06	–0·13, 0·00	–0·18	0·065	0·042	–0·015	–0·06	–0·13, 0·02	–0·16	0·1

UIC: urinary iodine concentration; TSH: thyroid-stimulating hormone; T4: thyroxine; PSS: perceived stress scale.

*P*-values in bold were statistically significant.

The dependent variable was perceived stress or hair cortisol (pg/mg). The independent variables were iodine/thyroid status indicators.

* Model I: controlled for sex, age, educational level and BMI-for-age z-scores.

† Model II: controlled for sex, age, educational level, BMI-for-age z-scores and antidepressant use.

## Discussion

In this case–control study, we found an adequate iodine status and no significant difference in UIC between Swiss adolescents with and without pMDD. We expected thyroid dysfunction, specifically hypothyroidism or subclinical hypothyroidism, to be associated with pMDD, and although we could not confirm this hypothesis, we found significantly lower TSH and FT4 levels among cases compared to controls. Against our hypothesis of inadequate iodine nutrition to be associated with aberrant thyroid parameters in pMDD, we found a trend for lower TSH values among cases with antidepressant treatment. Furthermore, we found a significantly higher prevalence of isolated hypothyroxinaemia among cases (15 %) compared to controls (4 %), which mainly occurred in female adolescents. Finally, we found higher PSS scores but lower hair cortisol levels among cases compared to controls, with a trend for lower hair cortisol levels in females but not in males.

In this study, we found lower TSH and FT4 levels as well as a higher prevalence of hypothyroxinaemia among depressed adolescents compared to healthy controls. Hypothyroxinaemia is a condition characterised by normal TSH and low thyroxine concentrations and has been described predominantly in iodine deficiency during pregnancy^(56)^. Hypothyroxinaemia during pregnancy has been associated with worse developmental outcome of the offspring^(57)^. The UIC did not differ significantly between cases and controls in this study. Furthermore, UIC for both groups was within the recommended UIC reference range for adequate iodine nutrition of 100–199 µg/l among adolescents^(44)^. This finding suggests appropriate iodine nutrition among the adolescents. Therefore, the observed lower TSH and FT4 levels, as well as higher prevalence of hypothyroxinaemia among the cases, might be caused by other factors rather than through low iodine intake. Among Polish women of childbearing age, vitamin D deficiency, insulin resistance, increased BMI and an abnormal lipid profile were associated with increased odds for hypothyroxinaemia^(58)^. Among Chinese pregnant and non-pregnant women, hypothyroxinaemia was higher among women with iron deficiency compared to women without iron deficiency^(59)^. Interactions between iron and iodine have been shown in rats, where iron deficiency anaemia reduced the activity of thyroid peroxidase, a heme-containing enzyme catalysing the first two steps of thyroid hormone synthesis^(60)^. In our study, differences in thyroid status parameters could not be explained by iron status (results not shown). However, iron status in this study sample might have been blunted by a higher proportion of cases receiving iron treatment (results not shown). Finally, among Chinese adult MDD patients, the use of mirtazapine, an antidepressant involving adrenoceptors and 5-hydoxytryptamine receptors, was associated with an increased risk for hypothyroxinaemia^(55)^. In our study, we observed a trend for lower TSH values among cases with current use of antidepressants compared to cases without current antidepressant use, but FT4 levels did not differ between antidepressant users and non-users. Thus, the use of antidepressants may only partly explain the observed aberrant thyroid hormone levels among cases compared to controls. Overall, the increased prevalence of hypothyroxinaemia among depressed adolescents compared to healthy controls may be explained by several factors, including interactions with other nutrients such as iron, TAG, antidepressant use or increased conversion of thyroxine to triiodothyronine without feedback on TSH. To confirm possible interactions between nutrients or medication, further research is needed.

Stress hormones known as glucocorticoids and primarily cortisol are known to increase during stress situations^(61)^. Against our hypothesis, we found lower hair cortisol levels among cases compared to controls. However, these data contribute to ambiguous evidence of hair cortisol levels in depressive disorders, with some other studies also finding increased hair cortisol levels in depressed individuals compared to controls^(62)^. Therefore, these results on hair cortisol levels should be interpreted with caution in the context of depressive disorders. When using the adolescent’s perceived stress as an approximation to describing the HPA axis, higher perceived stress scores were associated with higher odds for depression. Further, although not significant, a negative trend between thyroxine levels and PSS scores could be observed. This finding supports the hypothesis of a link between the HPT and the HPA axis being involved in the aetiology of depression; however, this hypothesis warrants further investigation in adolescents^(25)^.

This study has several strengths and limitations. First, our study population consists of well-matched participants with and without pMDD. Also, for cases, only individuals with a clinical diagnosis of pMDD of moderate to severe depressive symptoms were enrolled on the study. Next, we assessed the adolescent depression score using an interviewer-administered assessment which includes non-verbal items, allowing a comprehensive assessment of the severity of the adolescent’s depression. Despite the strength of our study design, due to its observational nature, no causal conclusions can be drawn. Further, one limitation of this study was that the iodine status of the participants was determined based on only one single spot urine sample. According to previous results from our group, to calculate the UIC on an individual level with a 20 % precision, at least ten spot urine samples are needed^(63)^. An additional limitation of this study is that we did not measure free triiodothyronine concentrations, and we did not assess thyroid autoantibodies; these would have allowed us to better characterise thyroid dysfunction. Therefore, results from the regression analyses including spot UIC have to be interpreted with caution. Nevertheless, the WHO states that casual single urine samples can provide an adequate assessment of iodine nutrition on a population level^(64)^. Therefore, the combination of UIC measurements with FT4 and TSH concentrations within this sample is valuable as it helps to interpret the origin of the observed thyroid dysfunctions. Finally, the results on cortisol concentrations were only obtained from a sub-sample of participants and therefore need to be interpreted with caution.

In conclusion, we found a higher prevalence of hypothyroxinaemia in adolescents with a diagnosis of depression compared to matched healthy controls. Also, our results suggest a link between hypothyroxinaemia and pMDD unrelated to iodine status or iron deficiency. Potential risk factors for hypothyroxinaemia and aberrant thyroid hormone parameters among adolescents, especially in females, with pMDD should be further investigated also in the context of antidepressant use.
